# Maternal Lineage of Warmblood Mares Contributes to Variation of Gestation Length and Bias of Foal Sex Ratio

**DOI:** 10.1371/journal.pone.0139358

**Published:** 2015-10-05

**Authors:** J. Kuhl, K. F. Stock, M. Wulf, C. Aurich

**Affiliations:** 1 Graf Lehndorff-Institut for Equine Science, Vetmeduni Vienna, Neustadt (Dosse), Germany; 2 Artificial Insemination and Embryo Transfer, Vetmeduni Vienna, Vienna, Austria; 3 Vereinigte Informationssysteme Tierhaltung w.V. (vit), Verden (Aller), Germany; University of Tasmania, AUSTRALIA

## Abstract

Maternal lineage influences performance traits in horses. This is probably caused by differences in mitochondrial DNA (mtDNA) transferred to the offspring via the oocyte. In the present study, we investigated if reproductive traits with high variability—gestation length and fetal sex ratio—are influenced by maternal lineage. Data from 142 Warmblood mares from the Brandenburg State Stud at Neustadt (Dosse), Germany, were available for the study. Mares were grouped according to their maternal lineage. Influences on the reproduction parameters gestation length and sex ratio of offspring were analyzed by simple and multiple analyses of variance. A total of 786 cases were included. From the 142 mares, 119 were assigned to six maternal lineages with n≥10 mares per lineage, and 23 mares belonged to smaller maternal lineages. The mean number of live foals produced per mare was 4.6±3.6 (±SD). Live foal rate was 83.5%. Mean gestation length was 338.5±8.9 days (±SD) with a range of 313 to 370 days. Gestation length was affected by maternal lineage (p<0.001). Gestation length was also significantly influenced by the individual mare, age of the mare, year of breeding, month of breeding and sex of the foal (p<0.05). Of the 640 foals born alive at term, 48% were male and 52% female. Mare age group and maternal lineage significantly influenced the sex ratio of the foals (p<0.05). It is concluded that maternal lineage influences reproductive parameters with high variation such as gestation length and foal sex ratio in horses. In young primiparous and aged mares, the percentage of female offspring is higher than the expected 1:1 ratio.

## Introduction

Genetic and genomic studies mostly focus on the variation that can be traced back to chromosomal deoxyribonucleic acid (DNA) representing the main part of genetic material transferred to the next generation. However, the dam is contributing further genetic material to her offspring via mitochondrial DNA (mtDNA). Mitochondria in the conceptus are strictly inherited through the maternal lineage. In contrast, paternal mitochondria disintegrate soon after fertilization [1] and transmission of paternal mtDNA does virtually not exist [2]. As in other species [3–6], heterogeneity in mtDNA assists in disentangling the origins and geographical dispersal of populations in equines because mtDNA characterizes maternal lineages [7].

An influence of maternal lineage on racing performance in the Thoroughbred race horse has been demonstrated [8,9]; and a similar impact on performance traits has also been assumed for other horse breeds [10]. It is so far unknown if and how performance selection via maternal lineages affects reproductive traits in horses. Such effects may most likely be demonstrable for reproductive traits with high variability within the population such as gestation length and fetal sex ratio. Because both parameters vary considerable in horses, this species was chosen for the present investigation.

Gestation length in horses can vary between 335 and 345 days, but viable foals are also born with extreme gestation lengths between 320 and 360 days [11–15]. Therefore when gestation length is >340 days and mares do not conceive before the second postpartum ovulation, i.e. 30 days after foaling, the date of parturition will be gradually postponed with every year, resulting in increasingly late birth of offspring. Loss of synchronization between the time of foaling and optimal environmental conditions will impair survival and development of the neonate in non-managed, i.e. wild and feral horses. In stabled or captured horses, more accurate prediction of parturition in mares would contribute to prevention of foal loss because early recognition of a foaling problem and rapid, appropriate intervention are critical to the survival of a foal [16]. A more profound knowledge on factors that influence gestation length in horses is therefore desirable. Gestation length is already known to be affected by exogenous factors such as year or month of breeding, but also by various endogenous factors like age and parity of the dam, and the sex of the fetus (reviewed by [14]). In addition, considerable individual differences among mares have been described [17], and heritability of gestation length was considered high enough to respond to selection (Valera et al., 2006 [17]: h^2^ = 0.21; Langlois and Blouin, 2012 [18]: h^2^ = 0.08–0.16). The influence of maternal lineage on gestation length in horses has not been investigated so far.

Considerable variation has also been reported for the fetal sex ratio in the horse, with both environmental and genetic influences. Differences in nutritional status can shift the percentage of male foals from as low as 3% in mares losing body condition at the time of conception to 80% when mares gained weight at conception [19]. In the horse, early conceptus loss is relatively high and seems to contribute to preference of one or the other sex apparently because female conceptuses have a better chance to survive suboptimal intrauterine conditions (reviewed by [20]). On the other hand, mitochondrial dysfunction or abnormalities in the oocyte contribute to conceptus loss and may at least in part be caused by defects at the mtDNA level [1].

The aim of the present study was analyzing differences in gestation length and fetal sex ratio among mares kept under identical management and environmental conditions, with particular focus on the maternal lineage of the mares. Considering several factors of potential relevance for these traits, we followed the hypothesis that maternal lineage influences gestation length and sex ratio of offspring in the horse.

## Materials and Methods

### Animals and experimental design

Breeding records of mares from the Brandenburg State Stud at Neustadt (Dosse), Germany, were analyzed retrospectively for this study. No specific permissions were required for the activities with the animals (horses) as all animal work consisted of routine breeding management at the Brandenburg State Stud and was not conducted for experimental purposes. Pedigree information of all mares was made available by the Brandenburg-Anhalt Breed registry, Neustadt (Dosse), through the central animal data base at Vereinigte Informationssysteme Tierhaltung w.V., Verden (Aller), Germany.

The stud keeps a broodmare herd of approximately 40 animals with the aim to annually produce one foal per mare. Mare lineage has been continuously recorded since 1946, and for some mares can be traced back until foundation of the stud in 1788. Data from 142 Warmblood mares including their breeding history were available for the study beginning with the breeding season 1992 and lasting until 2011. In 1992, ultrasound examination of the genital tract was introduced into the stud’s breeding management and the exact day of ovulation was always reliably determined.

In the stud, mares are entered into the broodmare herd as maiden mares at the age of three years. If mares do not fulfil the expectations of the stud management with regard to quality of the offspring or fertility for more than one consecutive year, they are mostly excluded from the herd and sold. Only mares meeting these requirements were included into the study.

At the stud farm, the breeding season lasts from 1 February to 15 June. During this time, mares are monitored for signs of estrus every other day. When a mare displays estrous behavior, she is referred to the stud’s veterinarian. Mares are assigned for breeding if a preovulatory follicle together with endometrial edema is detected by gynecological examination including transrectal ultrasound. Mares are inseminated with raw or cooled-shipped semen or in exceptional cases also bred by natural covering every other day until detection of a corpus luteum (Day 0). If mares are inseminated with frozen semen this is done within 6 hours after detection of a corpus luteum. Pregnancy diagnosis is performed between days 14 and 18 after ovulation and repeated at approximately day 35 by transrectal ultrasound. For the study, gestation length was calculated from the day of ovulation until the day of parturition of a viable foal. In case of pregnancy loss, abortion or stillbirth, data were excluded from analysis.

Using maximum depth of the pedigrees, the proportions of genes contributed by different breeds or breed groups were calculated on the basis of the breed specifications in the pedigree data. Inbreeding coefficients were determined using the software PEDIG [21]. For all mares, pedigree information was complete over the first four ancestral generations, decreased from 98% in the fifth to 67% in the tenth, and was still > 50% in the 25^th^ generation. The mares of the stud were grouped according to their maternal lineage, i.e. all mares directly descending from one founder mare were assigned to the same maternal lineage. On this basis, the 142 mares could be assigned to 13 mare families. The pedigree based affiliation of mares to the different maternal lineages was confirmed in a total of 38 active broodmares that were available at the stud in 2011 by mtDNA sequencing (results not shown). Only maternal lineages that consisted of at least ten mares were considered individually in the subsequent analyses (i.e. lineages 1, 2, 6, 7 and 8; n = 119; Table 1).

**Table 1:** Distribution of data among the maternal lineages. Numbers of Warmblood mares, breeding records (breeding seasons x mares) and pregnancies that resulted in birth of a living foal and their affiliation to the five individual mare families with more than 10 members.

Stratification variable	Maternal lineage					Total
	1	2	6	7	8	
**Mares**	46	27	10	17	19	**119**
**Breeding records**	272	161	39	95	89	**656**
**Foals**	220	130	35	82	71	**538**

### Statistical Analysis

Influences on the reproduction parameters gestation length and sex ratio of offspring were analyzed by simple and multiple analyses of variance using the procedures GLM and MIXED of the SAS software package, version 9.2 (Statistical Analysis System; SAS Institute Inc., Cary, NC, USA, 2014). In each case, the reproduction parameters (gestation length in days and foal sex with repeated observations, and the proportion of male foals with single observations per mare) were considered as dependent variables, and potential influencing factors were considered individually and in different combinations as independent variables. From the breeding records (N = 786) and the basal data of the mares, information on the mare's age at breeding, year and month of breeding, foal sex, and the size of the mare (height at withers as measured at the time of studbook entry) were available. From the pedigree analyses, inbreeding coefficients and gene proportions were further accessible for all mares. According to the data structure and to maximize power of the statistical analyses, class variables were derived and used for the modelling where applicable. To identify possibly co-linearity among the factors to be tested, extensive cross distribution analyses were performed. This allowed ensuring that only mutually independent variables were jointly considered in the model.

Choice of models was based on model fit statistics and performed in two steps. First, fixed effects and interactions were tested singularly and jointly with GLM. The multiple alternative models were compared with the respective full model to identify the most parsimonious one which still captured the structure of our data. Selection criteria were the proportion of explained variance and the results of likelihood ratio tests and only effects with error probabilities of P < 0.05 were retained. To account for the repeated observations per mare, the individual mare was subsequently added as random effect in the final analyses which were performed with MIXED. The inconsistent use of sires interfered with their individual consideration in the statistical analyses (viable foals descending from 188 sires with on average 3.4 foals in the data, 36% of these sires represented with single foal, 82% with less than five foals) and was therefore not considered.

Statistical models were as follows:

Model A (gestation length; 640 breeding records):
  $$y_{ijklmnpq}=\mu +Age_{i}+BYear_{j}+BMonth_{k}+MLine_{l}+FSex_{m}+PBreed_{n}+mare_{p}+e_{ijklmnpq}$$
Model B (foal sex; 650 breeding records):
  $$y_{ijklopq}=\mu +Age_{i}+BYear_{j}+BMonth_{k}+MLine_{l}+Size_{o}+mare_{p}+e_{ijklopq}$$

with

y_i…q_ = gestation length (in days) or foal sex (1 = male, 0 = female) of the i…q-th breeding record,

μ = model constant (population sample average),

Age_i_ = fixed effect of the i-th age at breeding (i = 1–4; 3 years, 4 to 8 years, 9 to 12 years, >12 years),

BYear_j_ = fixed effect of the j-th year of breeding (j = 1–5; before 1995, 1996–1999, 2000–2003, 2004–2007, 2008–2011),

BMonth_k_ = fixed effect of the k-th month of breeding (k = 1–4; March, April, May, June to February),

MLine_l_ = fixed effect of the l-th maternal lineage (l = 1–6; 5 individual mare families plus remaining herd),

FSex_m_ = fixed effect of the m-th sex of the foal (m = 1–2; male, female),

PBreed_n_ = fixed effect of the n-th proportion of Arabian and Thoroughbred genes (n = 1–2; < 25%, ≥ 25%),

Size_o_ = fixed effect of the o-th size of the mare (o = 1–3; 160–163 cm, 164–166 cm, 167–176 cm),

mare_p_ = random effect of the p-th mare (p = 1–139),

e_i…q_ = random residual.

Data are given as least square means (LSM) ± standard error of mean (SEM) unless otherwise indicated. An error probability (p-value) <0.05 was considered significant. Because of the consistency of the results obtained with the different models in the simple and multiple ANOVA, only the results obtained with the final models and the procedure MIXED are reported.

## Results

**Fig 1:**
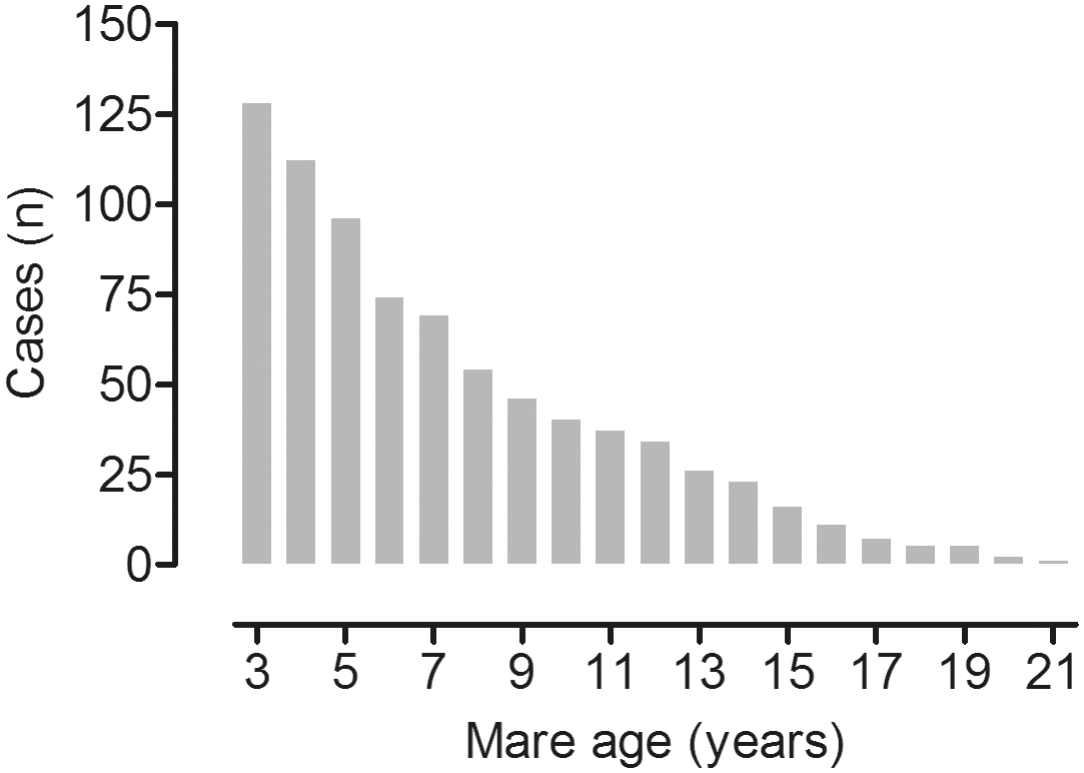
Mare age at breeding. Distribution of cases included into the analysis (n = 786) in relation to the age of the respective mare at breeding—mares (n = 142) were bred for the first time at the age of 3y and remained within the herd for up to 19 years.

Pedigree information was 100% complete for at least 4 ancestor generations and 56% for the 20^th^ generation. Mean (±SD) degree of inbreeding was 0.31±0.57%, with a maximum of 2.63%. The mare population did not differ in its composition with regard to maternal lineages during the study period. The mares were used for breeding between one and nineteen consecutive breeding years (5.4±4.4 breeding years per mare; mean±SD; Fig 1). The mean number of live foals produced per mare was 4.6±3.6 (±SD) with a maximum of 15 live foals produced in one mare. Live foal rate was 83.5% (640 pregnancies with live foals in 786 breeding seasons). In a total of 127 cases, the mare either did not become pregnant or lost her pregnancy (abortion or birth of a non-viable foal). In 14 cases (12 mares in one year and one mare in two years), mares were not bred due to management reasons, 4 mares died during the respective breeding seasons.

**Fig 2:**
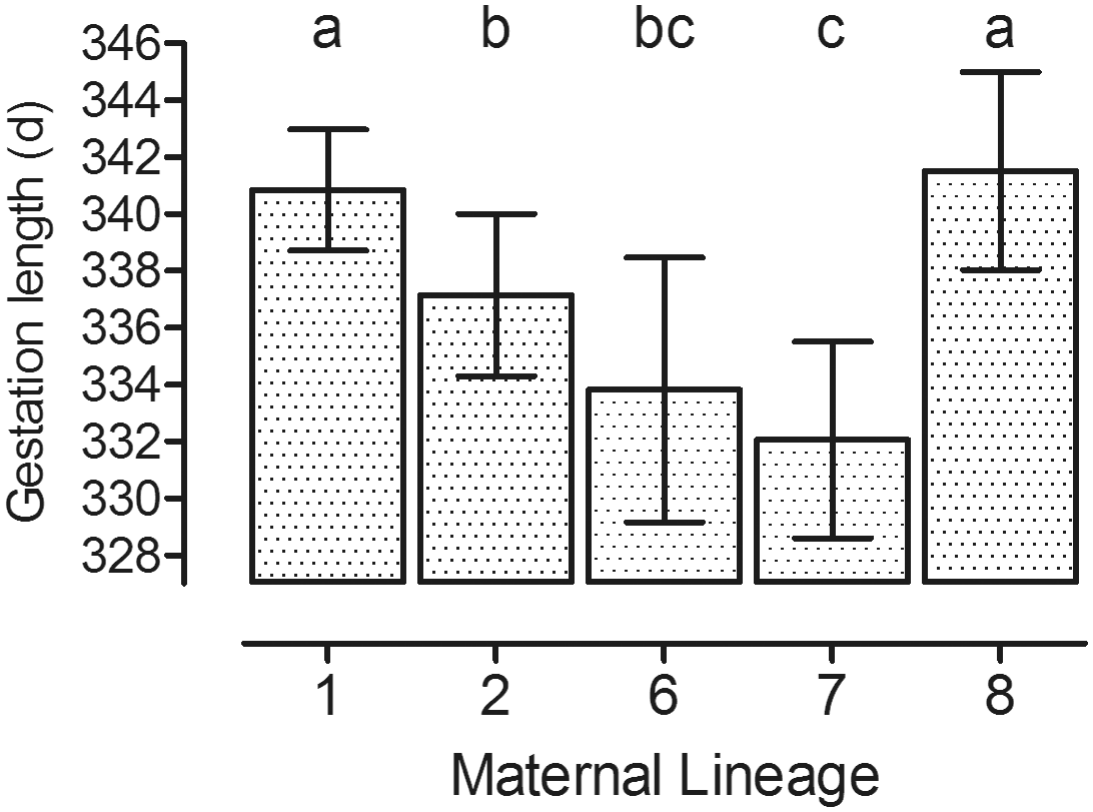
Influence of maternal lineage on gestation length. Maternal lineage (1: n = 220; 2: n = 130; 6: n = 35; 7: n = 82; 8: n = 71) influences gestation length (Model A p<0.001; abc: groups with different superscript letters differ significantly; p<0.05; LSM ± upper and lower confidence limits).

**Fig 3:**
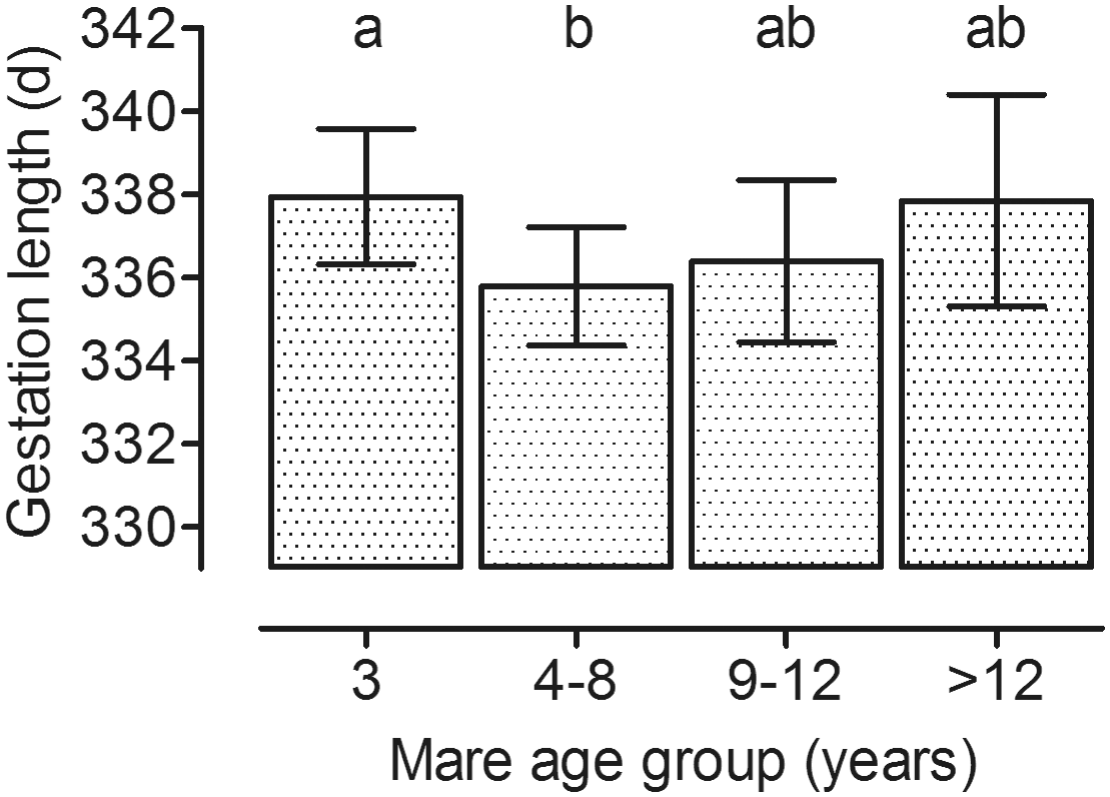
Influence of mare age at conception on gestation length. Gestation length in mares is influenced by mare age at conception (Model A p = 0.005; groups: 3 years: n = 116, 4–8 years: n = 337, 9–12 years: n = 124, >12 years: n = 63; ab: groups with different superscript letters differ significantly; p<0.05; LSM ± upper and lower confidence limits).

Mean gestation length (±SD) was 338.5±8.9 days, with a range of 313 to 370 days. Gestation length was affected by maternal lineage (Model A, p<0.001) and was longer in mares of lineages 1 and 8 than in all other maternal lineages (p< 0.05; Fig 2). Gestation length was also significantly influenced by the individual mare, age of the mare (Model A, p = 0.005; Fig 3), year of breeding, month of breeding and sex of the foal but not by the percentage of Arab and Thoroughbred ancestors in the pedigree (Model A; Table 2; S1 Table). Gestation length increased from the beginning until the end of the period evaluated (before 1995: 336.0±1.4 d; 1996–1999: 335.9±1.1 d; 2000–2003: 337.1±0.9 d; 2004–2007: 336.7±0.9 d; 2008–2011: 339.2±0.9 d; Model A, p = 0.0243). It was also influenced by the time of the year the mares were bred (March: 339.7±0.9 d; April: 338.7±0.8 d; May: 336.8±0.9 d; June-February: 332.9±1.0 d; Model A, p<0.0001). Gestation length for male foals (338.3±0.8 d) was longer than for female foals (335.7±0.8 d; Model A, p<0.001).

**Table 2:** Influence of different factors on gestation length (n = 640 pregnancies) in Warmblood mares (n = 142).

Model A		
*Mixed—Covariance Parameter Estimates*		
*Explanatory variables*	*Z*	*P*
Mare	5.93	<0.001
*Mixed—Type 3 Tests of Fixed Effects*		
*Explanatory variables*	*F*	*P*
Maternal lineage	5.79	<0.001
Age of mare	4.30	0.005
Year of breeding	2.83	0.024
Month of breeding	21.06	<0.001
Sex of the foal	24.00	<0.001
Percentage of Arabian and Thoroughbred ancestors in pedigree	0.79	0.375

**Fig 4:**
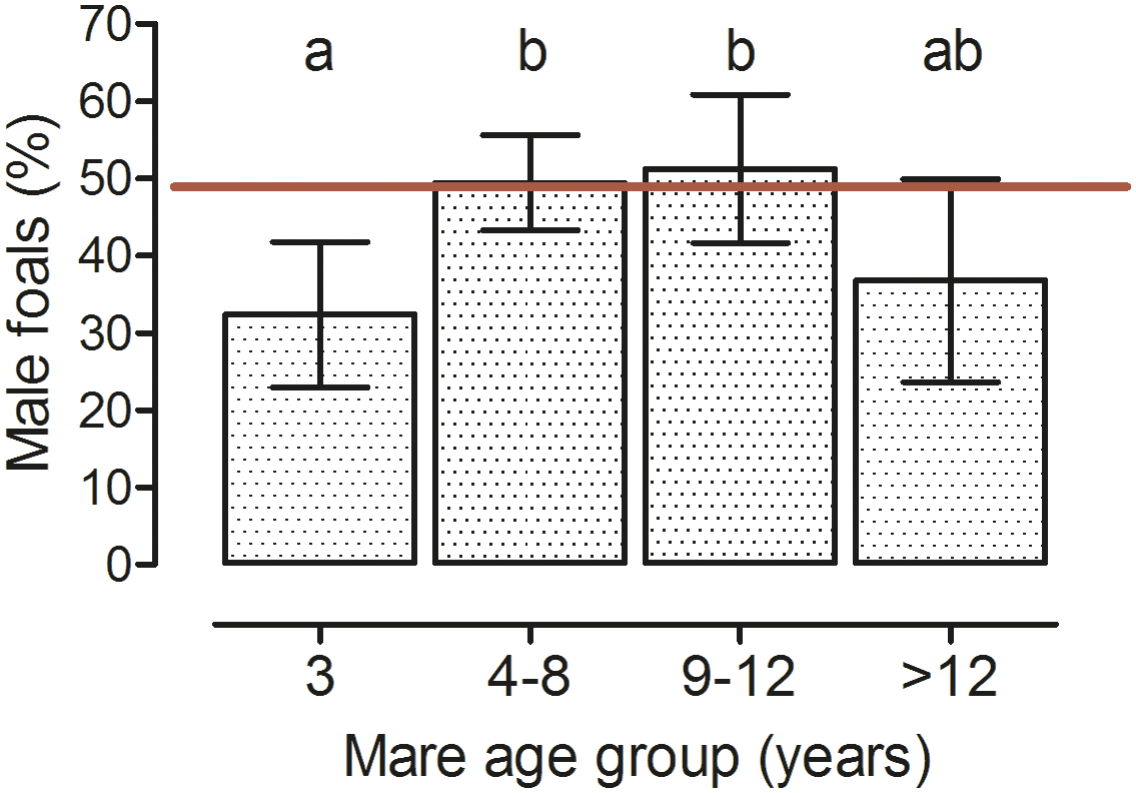
Percentage of male foals among the offspring of mares from different age groups. Mare age at conception has an impact on the proportion of fillies and colts born (Model B, p = 0.003; 3 years: n = 116, 4–8 years: n = 338, 9–12 years: n = 124, >12 years: n = 63; LSM ± upper and lower confidence limits; ab: groups with different superscript letters differ significantly, p<0.05; red horizontal line indicates expected 50% ratio of male foals born).

**Fig 5:**
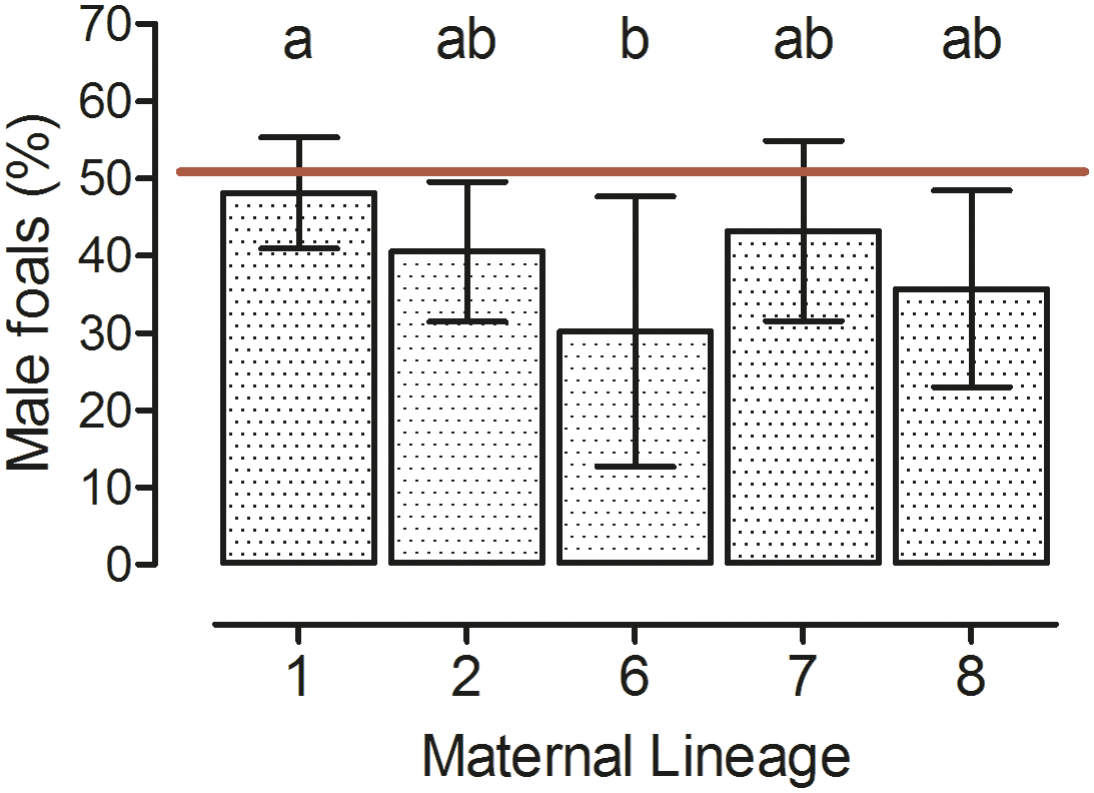
Percentage of male foals among the offspring of mares from different maternal lineages. Maternal lineage has an impact on the proportion of fillies and colts born (Model B, p = 0.027). Foals from maternal lineages: 1: n = 220, 2: n = 130, 6: n = 35, 7: n = 82, 8: n = 71 (LSM ± upper and lower limit of confidence; ab: groups with different superscript letters differ significantly, p = 0.0459; red horizontal line indicates expected 50% ratio of male foals born).

Of the 640 foals born alive at term, 48% (310) were male and 52% (330) female with no significant deviation from an expected 1:1 sex ratio. Mare age group and maternal lineage significantly influenced the sex ratio of the foals (Model B, p<0.05; Table 3; S2 Table). Differences for mares grouped by age and maternal lineage are shown in Figs 4 and 5, respectively.

**Table 3:** Influence of different factors on foal sex ratio (n = 640 pregnancies) in Warmblood mares (n = 142).

Model B		
*Mixed—Covariance Parameter Estimates*		
*Explanatory variables*	*Z*	*P*
Mare	0.26	0.399
*Mixed—Type 3 Tests of Fixed Effects*		
*Explanatory variables*	*F*	*P*
Maternal lineage	2.56	0.027
Age of mare	4.70	0.003
Year of breeding	1.37	0.243
Month of breeding	0.48	0.698
Mare size	1.82	0.164

## Discussion

In the present study maternal lineage significantly influenced gestation length and foal sex ratio in horses. To the best of our knowledge, this has neither been proven for the horse nor for other mammalian species so far. In the horse, gestation length is highly variable. Selection against maternal lineages with prolonged gestation length could be an interesting approach to avoid the gradual postponement of the birth date of offspring in mares with every year. The mare explains 13 to 18% of this variation whereas the sire contributes only 2 to 3% [18]. The maternal effect consists of two components, maternal genetics and environment [18]. Maternal genetics results from both chromosomal and mitochondrial DNA. Mitochondria in the fetus and its membranes exclusively originate from the mother because paternal mitochondria are destroyed after fertilization [1]. Mitochondrial function depends on the coordinated expression of nuclear and mitochondrial genomes. Mutation of mtDNA is responsible for the development of mitochondrial haplogroups which can modulate mitochondrial metabolism, cell respiration and defense mechanisms against reactive oxygen species (reviewed by [20]). Maternal lineages of the mare population involved in the present study are clearly distinguishable by differences in the sequence of their mtDNA (data not shown). In two of the haplogroups mean gestation length was significantly prolonged which is suggested to be a result of differences in mitochondrial function.

The effect of mare age on gestation length is controversially discussed. While a linear increase in gestation length with maternal age has been reported [18], differences in gestation length with regard to age were not found in other studies [11,13]. In the present investigation, a longer mean gestation length was found in three year-old maiden (i.e. primiparous) mares and in aged mares, but only when the mare itself was added as a random factor into the statistical model. It is suggested that differences in gestation length between young primiparous and aged pluriparous mares on the one side and mature pluriparous mares on the other side depend on differences in endometrial function. In primiparous (maiden) mares, the fetomaternal contact area of the placenta as well as the surface density of chorionic microcotyledons is smaller than in multiparous mares [22]. A similar picture as in maiden mares exists in mares older than 15 years. A less optimal development of the placenta contributes to smaller fetuses in these two mare groups [22]. Detrimental effects of a less-developed placenta are likely to also increase gestation length.

Similarly, in the present study, in maiden and aged mares the sex ratio of foals markedly deviated from the expected 1:1 ratio with a higher percentage of female offspring in these two categories. Hence, breeding maiden and aged mares should not be done with the aim to produce male offspring because the sex ratio might be female-biased in these mares. In the horse, the uterine environment at conception and during early pregnancy has a pronounced effect on sex ratio of the offspring at birth (reviewed by [20]). Findings of the present study are thus in agreement with the hypothesis that the early female horse conceptus has mechanisms that counteract its death under detrimental uterine conditions [20,23]. In further support of this suggestion, a better placental adaptation to adverse conditions in female than in male pregnancies has been postulated [24]. Therefore, on average a higher proportion of female foals has to be expected from maiden or aged mares. Influences of maternal lineage on fetal sex ratio in the present study suggest that mtDNA haplotype also affects early sex-specific conceptus development and endometrial function which is in agreement with studies in ruminants [25–27] and has already been suggested for the horse [28]. In agreement with the hypothesis by Trivers and Willard [29], variations in sex ratio are often related to food availability and condition of the mother at conception [30]. Camargue mares kept under semi-feral conditions [31] and feral horses in New Zealand [32] produced an increased percentage of female foals following breeding seasons with poor food availability. In contrast, in the Warmblood mares in our study no effect of year of breeding on foal sex ratio could be detected under intensively managed stud farm conditions. Feeding in these mares is adjusted to maintain body condition, i.e. poorer pasture quality is compensated by addition of hay or concentrates to the mares’ daily feeding ration. An age related bias of sex ratio towards females in the progeny of younger and aged mares as in the present study is in agreement with findings from different wild ass subspecies [33] but not in feral or semi-feral horses [31,34]. However, in these studies the number of primiparous mares was low, they were not intensively managed and were kept under varying environmental conditions.

The gestation length in the Warmblood mares of our study showed a range of almost two months for live foals. Effects of month of breeding and sex of the foal on gestation length reported previously [12,16,17] could be confirmed. Although the relatively short breeding period used on the stud with the majority of mares bred within an interval of three months reduced the occurrence of seasonal effects, gestation length was shorter when mares were bred very early or late in the year. The fact that less fertile, i.e. less productive mares are sometimes excluded from the herd may have biased the results of the present study in a balancing, but not in an exaggeratory way. Many of these mares are excluded because they do not conceive within the relative short breeding season of the stud. This often affects mares with above-average gestation length foaling late in the year. Therefore, in many cases, low fertility is not based on reproductive problems but on the fact that a mare is not pregnant at the end of the season. If these mares had been remained in the herd, the number of cases with above-average gestation length might have been even higher.

Even though the mean duration of pregnancies producing male foals was longer than pregnancies resulting in birth of female foals, the range of gestation length was still large for both sexes. In contrast to the suggestion by Langlois and Blouin [18], determination of foal sex during pregnancy [20] is therefore not helpful for prediction of pregnancy length in the horse. In addition, prediction of gestation length based on the average of previous pregnancies as suggested in a study on Draft mares [35] does not appear sensible enough because in the present population in six mares that carried at least 12 foals to term, gestation length varied by 16 to 35 days in individual mares.

In the present study, gestation length increased from the beginning to the end of the observation period. At present we cannot satisfyingly explain this finding. During this time period, the mares were mainly bred by artificial insemination, the percentage of mares mated by natural cover was constantly low and could thus not contribute to this development. The level of inbreeding of the Warmblood mares in the present study is similar to the mare population in France [18] or in Hanoverian Warmblood mares [36], but much lower than in Standardbred trotters or Finnhorses [37]. The degree of inbreeding is therefore unlikely to contribute to this increase in gestation length. Because Arabian as well as Thoroughbred mares have been reported to have longer mean gestation lengths than mares from other breeds kept under similar conditions (e.g. [11,17]), we tested if the percentage of Arabian and Thoroughbred ancestors in the pedigree of the mares influenced gestation length but this hypothesis was not supported.

## Supporting Information

S1 Table Influence of different factors on gestation length in Warmblood mares. Fixed effects model A—raw means.
(PDF)

S2 Table Influence of different factors on foal sex ratio in Warmblood mares. Fixed effects model B—raw means.
(PDF)

## References

[1] Van Blerkom J. Mitochondria in human oogenesis and preimplantation embryogenesis: engines of metabolism, ionic regulation and developmental competence. Reproduction. 2004 9;128(3):269–80. 15333778 10.1530/rep.1.00240

[2] Taylor RW, McDonnell MT, Blakely EL, Chinnery PF, Taylor GA, Howell N et al Genotypes from patients indicate no paternal mitochondrial DNA contribution. Ann Neurol. 2003 10;54(4):521–4. 14520666 10.1002/ana.10673

[3] Giuffra E, Kijas JM, Amarger V, Carlborg O, Jeon JT, Andersson L. The origin of the domestic pig: independent domestication and subsequent introgression. Genetics. 2000 4;154(4):1785–91. 10747069 10.1093/genetics/154.4.1785 PMC1461048

[4] Tapio M, Marzanov N, Ozerov M, Cinkulov M, Gonzarenko G, Kiselyova T et al Sheep mitochondrial DNA variation in European, Caucasian, and Central Asian areas. Mol Biol Evol. 2006 9;23(9):1776–83. 16782761 10.1093/molbev/msl043

[5] Naderi S, Rezaei HR, Taberlet P, Zundel S, Rafat SA, Naghash HR et al Large-scale mitochondrial DNA analysis of the domestic goat reveals six haplogroups with high diversity. PLoS One. 2007 10 10;2(10):e1012 17925860 10.1371/journal.pone.0001012 PMC1995761

[6] Edwards CJ, Bollongino R, Scheu A, Chamberlain A, Tresset A, Vigne JD et al Mitochondrial DNA analysis shows a Near Eastern Neolithic origin for domestic cattle and no indication of domestication of European aurochs. Proc Biol Sci. 2007 6 7;274(1616):1377–85. 17412685 10.1098/rspb.2007.0020 PMC2176208

[7] Cieslak M, Pruvost M, Benecke N, Hofreiter M, Morales A, Reissmann M et al Origin and history of mitochondrial DNA lineages in domestic horses. PLoS One. 2010 12 20;5(12):e15311 10.1371/journal.pone.0015311 21187961 PMC3004868

[8] Lowe CB. Breeding racehorses by the figure system. (ed. by Allison W). The Field and Queen (Horace Cox) Ltd, UK; 1895.

[9] Harrison SP, Turrion-Gomez JL. Mitochondrial DNA: an important female contribution to thoroughbred racehorse performance. Mitochondrion. 2006 4;6(2):53–63. 16516561 10.1016/j.mito.2006.01.002

[10] Hector C. Quaterback and Neustadt—something old, something new In: The making of the modern Warmblood—From Gotthard to Gribaldi. Sporthorse International Richmond South. Australia ISBN 978-0-646-54051-1. 2010 pp. 80–87.

[11] Davies Morel MC, Newcombe JR, Holland SJ. Factors affecting gestation length in the Thoroughbred mare. Anim Reprod Sci. 2002 12 16;74(3–4):175–85. 12417119 10.1016/s0378-4320(02)00171-9

[12] Heidler B, Aurich JE, Pohl W, Aurich C. Body weight of mares and foals, estrous cycles and plasma glucose concentration in lactating and non-lactating Lipizzaner mares. Theriogenology. 2004 4 1;61(5):883–93. 14757474 10.1016/s0093-691x(03)00279-6

[13] Winter GHZ, Rubin MIB, De La Corte FD, Silva CAM. Gestational length and first postpartum ovulation of Criollo mares on a stud farm in Southern Brazil. J Equine Vet Sci. 2007 12:531–4.

[14] Satué K, Felipe M, Mota J, Muñoz A (2011): Factors influencing gestational length in mares: a review. Livest Sci. 2011 4; 136(2–3): 287–94.

[15] Nagel C, Erber R, Bergmaier C, Wulf M, Aurich J, Möstl E et al Cortisol and progestin release, heart rate and heart rate variability in the pregnant and postpartum mare, fetus and newborn foal. Theriogenology. 2012 9 1;78(4):759–67. 10.1016/j.theriogenology.2012.03.023 22626780

[16] McCue PM, Ferris RA. Parturition, dystocia and foal survival: a retrospective study of 1047 births. Equine Vet J Suppl. 2012 2;(41):22–5. 22594021 10.1111/j.2042-3306.2011.00476.x

[17] Valera M, Blesa F, Dos Santos R, Molina A. Genetic study of gestation length in Andalusian and Arabian mares. Anim Reprod Sci. 2006 9;95(1–2):75–96. 16271285 10.1016/j.anireprosci.2005.09.008

[18] Langlois B, Blouin C. Genetic parameters for gestation length in French horse breeds. Livest Sci. 2012 7;14682–3):133–9.

[19] Cameron EZ, Linklater WL. Extreme sex ratio variation in relation to change in body condition around conception. Biol Lett. 2007 8 22;3(4):395–7. 17439844 10.1098/rsbl.2007.0089 PMC2390657

[20] Aurich C, Schneider J. Sex determination in horses—Current status and future perspectives. Anim Reprod Sci. 2014 4;146(1–2):34–41. 10.1016/j.anireprosci.2014.01.014 24598214

[21] Boichard D. PEDIG: a fortran package for pedigree analysis suited for large populations. 7^th^ Worl Congress on Genetics Applied to Livestock Production, Montpellier, France, 2002, Communication N° 28–13.

[22] Wilsher S, Allen WR. The effects of maternal age and parity on placental and fetal development in the mare. Equine Vet J. 2003 7;35(5):476–83. 12875326 10.2746/042516403775600550

[23] Beckelmann J, Budik S, Helmreich M, Palm F, Walter I, Aurich C. Sex-dependent insulin like growth factor–1 expression in preattachment equine embryos. Theriogenology. 2013 1 1;79(1):193–9. 10.1016/j.theriogenology.2012.10.004 23122604

[24] Aiken CE, Ozanne SE. Sex differences in developmental programming models. Reproduction. 2013 1 8;145(1):R1–13. 10.1530/REP-11-0489 23081892

[25] Sutarno, Cummins JM, Greeff J, Lymbery AJ. Mitochondrial DNA polymorphisms and fertility in beef cattle. Theriogenology. 2002 4 1;57(6):1603–10. 12035972 10.1016/s0093-691x(02)00664-7

[26] Jiao F, Yan JB, Yang XY, Li H, Wang Q, Huang SZ et al Effect of oocyte mitochondrial DNA haplotype on bovine somatic cell nuclear transfer efficiency. Mol Reprod Dev. 2007 10;74(10):1278–86. 17290429 10.1002/mrd.20698

[27] Reicher S, Seroussi E, Weller JI, Rosov A, Gootwine E. Ovine mitochondrial DNA sequence variation and its association with production and reproduction traits within an Afec-Assaf flock. J Anim Sci. 2012 7;90(7):2084–91. 10.2527/jas.2011-4673 22266988

[28] Choi YH, Ritthaler J, Hinrichs K. Production of a mitochondrial-DNA identical cloned foal using oocytes recovered from immature follicles of selected mares. Theriogenology. 2014 8;82(3):411–7. 10.1016/j.theriogenology.2014.04.021 24888683

[29] Trivers RL, Willard DE. Natural selection of parental ability to vary the sex ratio of offspring. Science. 1973 1 5;179(4068):90–2. 4682135 10.1126/science.179.4068.90

[30] Cameron EZ. Facultative adjustment of mammalian sex ratios in support of the Trivers-Willard hypothesis: evidence for a mechanism. Proc Biol Sci. 2004 8 22;271(1549):1723–8. 15306293 10.1098/rspb.2004.2773 PMC1691777

[31] Monard AM, Duncan P, Fritz H, Feh C. Variations in the birth sex ratio and neonatal mortality in a natural herd of horses. Behav Ecol Sociobiol. 1997 41(4): 243–249.

[32] Cameron EZ, Linklater W, Stafford KJ, Veltman CJ. Birth sex ratios relate to mare condition at conception in Kaimanawa horses. Behavioral Ecology. 1999 10:472–475.

[33] Saltz D. Rubenstein DI. Population Dynamics of a Reintroduced Asiatic Wild Ass (Equus Hemionus) Herd. Ecological Applications. 1995 5:327–335.

[34] Cameron EZ, Linklater WL. Individual mares bias investment in sons and daughters in relation to their condition. Anim Behav. 2000 9;60(3):359–367. 11007645 10.1006/anbe.2000.1480

[35] Aoki T, Yamakawa K, Ishii M. Factors affecting gestation length in heavy draft mares. J Equine Sci. 2013 6;33(6):437–40.

[36] Hamann H, Distl O. Genetic variability in Hanoverian warmblood horses using pedigree analysis. J Anim Sci. 2008 7;86(7):1503–13. 10.2527/jas.2007-0382 18310493

[37] Sairanen J, Nivola K, Katila T, Virtala AM, Ojala M. Effects of inbreeding and other genetic components on equine fertility. Animal. 2009 12;3(12):1662–72. 10.1017/S1751731109990553 22443550

