# Supplementary material for: Maternal Lineage of Warmblood Mares Contributes to Variation of Gestation Length and Bias of Foal Sex Ratio
Source: PLoS One. 2015 Oct 5;10(10):e0139358. doi: 10.1371/journal.pone.0139358 (PMC4593555; doi:10.1371/journal.pone.0139358)
Supplement: S1 Table — Fixed effects model A—raw means. (PDF) [file pone.0139358.s001.pdf]

Gestation Length

Fixed Effects - Model A

**Maternal Lineage**	N	Mean	Std Dev	Minimum	Maximum
1	220	341.3	9.0	313	370
2	130	337.2	8.1	314	364
6	35	334.9	9.5	320	357
7	82	333.0	7.9	318	352
8	71	341.5	6.9	325	354
RH	102	337.5	8.4	315	357

**Age of Mare**	N	Mean	Std Dev	Minimum	Maximum
3 y	116	339.3	9.2	320	368
4-8 y	337	338.0	8.5	313	361
9-12 y	124	338.4	8.8	314	370
> 12 y	63	339.5	10.1	315	358

**Year of Breeding**	N	Mean	Std Dev	Minimum	Maximum
≤1995	114	336.4	8.4	315	353
1996-1999	114	337.1	7.3	320	355
2000-2003	126	338.4	9.4	313	368
2004-2007	129	339.0	9.2	318	361
2008-2011	157	340.5	9.2	314	370

**Month of Breeding**	N	Mean	Std Dev	Minimum	Maximum
March	148	339.4	9.2	318	368
April	232	339.3	8.8	320	370
May	163	338.1	8.9	313	364
Jun-Feb	97	335.6	7.9	314	354

**Sex of the Foal**	N	Mean	Std Dev	Minimum	Maximum
Colts	310	339.3	8.6	320	370
Fillies	330	337.6	9.1	313	368

Percentage of Arabian and Thoroughbred Ancestors in Pedigree

	N	Mean	Std Dev	Minimum	Maximum
<25%	362	337.1	8.6	313	370
≥25%	278	340.2	8.9	314	368

J. Kuhl, K.F. Stock, M. Wulf, C. Aurich
