# Supplementary material for: Maternal Lineage of Warmblood Mares Contributes to Variation of Gestation Length and Bias of Foal Sex Ratio
Source: PLoS One. 2015 Oct 5;10(10):e0139358. doi: 10.1371/journal.pone.0139358 (PMC4593555; doi:10.1371/journal.pone.0139358)
Supplement: S2 Table — Fixed effects model B—raw means. (PDF) [file pone.0139358.s002.pdf]

# Proportion of Male Foals

Fixed Effects - Model B

| **Maternal Lineage** | N | Mean | Std Dev | Minimum | Maximum |
|---|---|---|---|---|---|
| 1 | 220 | 0.509 | 0.501 | 0 | 1 |
| 2 | 130 | 0.444 | 0.499 | 0 | 1 |
| 6 | 35 | 0.314 | 0.471 | 0 | 1 |
| 7 | 82 | 0.464 | 0.502 | 0 | 1 |
| 8 | 71 | 0.394 | 0.492 | 0 | 1 |
| RH | 102 | 0.6 | 0.492 | 0 | 1 |

| **Age of Mare** | N | Mean | Std Dev | Minimum | Maximum |
|---|---|---|---|---|---|
| 3 y | 116 | 0.347 | 0.478 | 0 | 1 |
| 4-8 y | 337 | 0.523 | 0.5 | 0 | 1 |
| 9-12 y | 124 | 0.536 | 0.501 | 0 | 1 |
| > 12 y | 63 | 0.4 | 0.494 | 0 | 1 |

| **Year of Breeding** | N | Mean | Std Dev | Minimum | Maximum |
|---|---|---|---|---|---|
| ≤1995 | 114 | 0.542 | 0.500 | 0 | 1 |
| 1996-1999 | 114 | 0.500 | 0.502 | 0 | 1 |
| 2000-2003 | 126 | 0.435 | 0.498 | 0 | 1 |
| 2004-2007 | 129 | 0.523 | 0.501 | 0 | 1 |
| 2008-2011 | 157 | 0.427 | 0.496 | 0 | 1 |

| **Month of Breeding** | N | Mean | Std Dev | Minimum | Maximum |
|---|---|---|---|---|---|
| March | 148 | 0.46 | 0.5 | 0 | 1 |
| April | 232 | 0.511 | 0.501 | 0 | 1 |
| May | 163 | 0.462 | 0.5 | 0 | 1 |
| Jun-Feb | 97 | 0.48 | 0.502 | 0 | 1 |

| **Mare Size** | N | Mean | Std Dev | Minimum | Maximum |
|---|---|---|---|---|---|
| 160-163 cm | 137 | 0.504 | 0.502 | 0 | 1 |
| 164-166 cm | 302 | 0.503 | 0.501 | 0 | 1 |
| 167-176 cm | 213 | 0.437 | 0.497 | 0 | 1 |

J. Kuhl, K.F. Stock, M. Wulf, C. Aurich
